# The WHO 2022 Classification of Renal Neoplasms (5th Edition): Salient Updates

**DOI:** 10.7759/cureus.58470

**Published:** 2024-04-17

**Authors:** Parth R Goswami, Gyanendra Singh, Tarang Patel, Rushang Dave

**Affiliations:** 1 Pathology, All India Institute of Medical Sciences (AIIMS), Rajkot, Gujarat, IND; 2 Pathology, All India Institute of Medical Sciences (AIIMS), Rajkot, IND; 3 Pathology, Shantabaa Medical College and General Hospital, Amreli, IND

**Keywords:** eosinophilic vacuolated tumour, low-grade oncocytic tumour, eloc-mutated renal cell carcinoma, eosinophilic solid and cystic renal cell carcinoma, alk-rearranged renal cell carcinoma, tfe3-rearranged & tfeb-altered rcc, smarcb1-deficient renal medullary carcinoma, molecularly defined renal cell carcinoma, who 2022 classification

## Abstract

The first categorization for renal tumours was made by the WHO in 1981 and included only renal cell carcinoma (RCC). After that, classification was continuously altered over five decades. The WHO 2022 Classification of Urinary and Male Genital Tumours 2022 (5^th^ edition) is molecular-driven and contains major revisions compared to the earlier classification from 2016. This revised edition divided renal tumours into four major broad categories: clear cell renal tumours, papillary renal cell tumours, oncocytic and chromophobe renal tumours, and collecting duct tumours. 'Other renal tumours’ and ‘molecularly defined renal carcinomas' are two other categories that were also included. Transcription factor binding to IGHM enhancer 3 (TFE3)-rearranged, TFEB-altered, elongin C (ELOC)-mutated (formerly TCEB1)-mutated, fumarate hydratase (FH)-deficient, succinate dehydrogenase (SDH)-deficient, anaplastic lymphoma kinase (ALK)-rearranged, and SWI/SNF-related matrix-associated actin-dependent regulator of chromatin subfamily B member 1 (SMARCB1)-deficient renal cell carcinomas are molecularly defined entities. Eosinophilic vacuolated tumours and low-grade oncocytic tumours are classified as emerging entities. Molecularly characterized renal tumours include those with SMARCB1 deficiencies, TFE3 rearrangements, TFEB alterations, ALK rearrangements, ELOC mutations, etc. Thyroid-like follicular carcinoma, eosinophilic vacuolated tumour, and low-grade oncocytic tumour are a few emerging entities of renal tumours. Improved therapy targets for each kidney tumour can be achieved using immunohistochemistry (IHC) and molecular definition updates. This study aims to highlight new developments in the WHO 2022 categorization of renal tumours with regard to diagnostic, morphological, molecular, IHC, clinical, and prognostic updates.

## Introduction and background

The initial classification of renal tumours was established in 1981 by the WHO, focusing exclusively on renal cell carcinoma (RCC). Over five decades, there has been a continuous process of altering classifications. The previous classifications of RCC were established based on different parameters. These parameters include cytoplasmic features, such as clear cell carcinoma; anatomical location of the tumor, such as collecting duct and renal medullary carcinoma; predominating architectural features, such as papillary RCC; tumors with embryological parts, such as metanephric adenoma; genetic association, such as hereditary leiomyomatosis-related RCC; and molecular alterations, such as Xp11 translocation associated or microphthalmia (MIT) family translocation-associated RCC [1,2,3].

The WHO classification of 2004 introduced several molecular alterations associated with RCC, such as MIT family translocation carcinoma [1]. This third edition incorporated the introduction of 12 histopathological subtypes. Subsequently, the fourth edition, published in 2016, expanded upon this classification system by including 16 subtypes. The most recent edition, the fifth, published in 2022, further augmented the classification system by incorporating a total of 21 histopathological subtypes. The recently published fifth edition of the WHO classification of renal tumours is a comprehensive categorization system that incorporates several factors such as morphology, immunohistochemistry, molecular characteristics, clinical data, epidemiology, and prognostic information [2,4].

The Genitourinary Pathology Society has provided an update on renal tumours, which has also been incorporated into the WHO's classification for 2022 [5]. This fifth edition was compiled by a group of 181 authors, includes over 900 photos, and references about 3600 sources [6]. The objective of this study is to elucidate the recent advancements in diagnostic, morphological, molecular immunohistochemistry (IHC), clinical, and prognostic aspects of the WHO 2022 classification of renal cancers, with a focus on its comparison to the previous 2016 classification.

## Review

The major updates in the WHO 2022 (5th edition) classification in comparison to the 2016 (4th edition) classification

Renal tumors can be classified into several overarching categories, including clear cell renal tumors, papillary renal cell tumors, oncocytic renal cell tumors, chromophobe renal cell tumors, and collecting duct tumors. In the WHO 2022 classification of renal malignancies, three new entities have been included: elongin C (ELOC)-mutated renal cell tumors [7], eosinophilic solid cystic renal cell tumors [8], and anaplastic lymphoma kinase (ALK) rearranged renal cell tumors [9]. Several revisions have been made to the categorization of papillary RCC, clear cell papillary renal cell tumors, MIT family renal cell cancers, fumarate-deficient renal cell tumors, and medullary carcinoma in the WHO 2022 classification.

An illustrative instance of a distinct genetic anomaly detected in particular kidney cancers, the BRAF p.V600E mutations are found in notable cases of metanephric stromal tumors, metanephric adenoma, and metanephric adenofibroma [8,10]. The WHO's 2022 classification of kidney cancers incorporated the inclusion of The International Classification of Diseases for Oncology (ICD-O) topographical and ICD-O morphological coding, as well as tumour, node, metastasis (TNM) staging (8th edition), for urological malignancies. The diagnostic criteria for renal cancers in the WHO 2022 classification encompass both essential and desirable elements, which encompass key morphological, IHC, and molecular findings for each category of renal malignancies. This review will comprehensively examine the significant modifications in the classification of renal tumours according to the WHO in 2022, in comparison to the previous classification established in 2016, whenever relevant.

Papillary RCC

Type 1 and type 2 papillary RCC were historically used to categorize the disease. This subdivision is not advised in the revised WHO 2022 classification, which refers to type 1 RCC as 'classic renal cell carcinoma' [11]. Related entities of type 2 are now counted as separate entities. For example, RCCs with deficiencies in fumarase hydratase (FH), ALK rearrangement, acquired cystic disease association, and eosinophilic solid and cystic RCC have been reported [12].

In the WHO 2022 classification of papillary RCC, new morphological variations are included, such as papillary renal cell tumour with reverse polarity [13], Warthin-like RCC [14], biphasic hyalinizing [15], biphasic squamoid or alveolar [16], thyroid-like follicular carcinoma [17], and biphasic hyalinizing renal cell tumour [13]. Specific genetic mutations have been found in a small number of these cancers, namely GATA binding protein 3 (GATA3) and Kirsten rat sarcoma (KRAS) virus mutations are seen in papillary renal cell cancers with reverse polarity [13], neurofibromatosis type 2 (NF2) mutation is seen in biphasic hyalinizing psammomatous renal cell tumours [18], and EWSR1-PATZ1 fusion is seen in thyroid-like follicular (TLF)-RCC [19].

Clear cell papillary renal cell tumours

The clear cell papillary renal cell tumor was initially identified in individuals with end-stage renal illness [20] and subsequently in sporadic cases [21]. In contrast to clear cell RCC, clear cell papillary renal cell tumours do not consistently exhibit Von Hippel-Lindau (VHL) syndrome gene mutation and 3p gene deletion [22]. The gross characteristics of a tumour often encompass a distinct and well-defined mass, occasionally accompanied by the presence of cystic alterations. Under microscopic examination, the cellular structure exhibits distinct characteristics, such as a transparent cytoplasm and the presence of solid, tubular, and papillary formations. It has been noted that tumours exhibiting indolent characteristics have no signs of spreading. Therefore, the nomenclature for clear cell papillary carcinoma has been revised in the new WHO 2022 classification to clear cell papillary tumours [14,23].

The FH-deficient RCC

The tumour in question was classified as a type 2 papillary renal cell tumour in the 2004 WHO classification [1]. According to the 2016 WHO classification (4th edition), a condition known as hereditary leiomyoma (HL)-linked RCC was identified. This condition is characterized by the presence of uterine and cutaneous leiomyoma, which exhibit aggressive and metastatic tendencies [2]. Several investigations have reported the presence of FH protein deficiency in various types of renal cell cancer, including tubulocystic carcinoma with dedifferentiated foci, type 2 RCC, unclassified high-grade RCC, and collecting duct carcinoma [24-26]. Therefore, the term 'hereditary leiomyomatosis and renal cell cancer' (HLRCC) has been replaced by 'FH-deficient renal cell carcinoma' in the WHO 2022 classification [27]. The FH-deficient renal cell carcinoma exhibits a range of architectural features, including papillary, solid, tubulocystic, and cribriform, when observed under a microscope.

The SMARCB1-deficient renal medullary carcinoma

This neoplasm is predominantly seen in the renal medulla. The phenomenon of SWI/SNF-related matrix-associated actin-dependent regulator of chromatin subfamily B member 1 (SMARCB1) protein nuclear expression loss has been noted in cases of renal medullary cancer. Renal medullary cancer has been reclassified as SMARCB1-deficient renal medullary carcinoma, as indicated by multiple sources [28-31]. This tumour is characterized by its infrequency and aggressive nature and is frequently observed in individuals with sickle cell trait and hemoglobinopathy [32]. Certain subtypes of renal carcinoma also exhibit secondary loss of SMARCB1. For instance, the presence of clear cell renal carcinoma with sarcomatoid transformation and FH-deficient renal cell cancer has been reported [33].

The TFE3-rearranged and TFEB-altered RCC

In the WHO 2016 classification (4th edition), transcription factor binding to IGHM enhancer 3 (TFE3)-rearranged RCC and transcription factor EB (TFEB)-rearranged RCC were grouped under the 'MIT family RCC' category. Few studies, however, have observed that TFEB-amplified RCC is seen commonly in elderly patients and has an associated worse prognosis as compared to TFEB-rearranged RCC. Hence, in the WHO 2022 classification, the TFE3-rearranged and TFEB-altered RCC categories were separated, and the MIT category no longer exists. In addition, TFEB-altered RCC is again subdivided into TFEB-rearranged and TFEB-amplified RCC [34-36].

The TFE3-rearranged RCC shows xp11 translocation. It commonly involves multiple genes such as ASPSCR1, SFPQ, PRCC, GRIPAP1, etc. Hence, the diversity of morphology observed in TFE3-rearranged RCC includes perivascular epithelioid cell neoplasms (PEComa) with a lack of PAX8 expression. The fluorescence in situ hybridization (FISH) assay or RNA sequencing is used to demonstrate this gene rearrangement [37,38].

The TFEB-rearranged RCC involves translocation (6:11) with TFEB-MALAT1 gene fusion. This tumour shows indolent behaviour. The morphological pattern in this category includes oncocytic, papillary, epithelioid, basement membrane material nodules, etc. [34,39,40]. The TFEB-amplified RCC shows 6p21 amplification. Hence, TFEB gene overexpression, along with overexpression of VEGFA in this tumour is usually associated with a worse prognosis and is seen in older patients [41,42].

Eosinophilic solid and cystic RCC

A novel entity has been included in the WHO's 2022 categorization of kidney tumours [43]. This tumour exhibits a higher incidence in females, a non-uniform distribution, and a correlation with the tuberous sclerosis complex [44,45]. This tumour is characterized by its large size, the presence of solid-cystic components, the occurrence of bleeding, and necrosis. In solid regions, cells are typically organized in an acinar or nested manner, exhibiting eosinophilia and possessing ample cytoplasm. The cells that line the cystic area have a hobnail characteristic, characterized by the presence of purple granules in the cytoplasm. These granules result from the aggregation of rough endoplasmic reticulum [46,47]. The IHC analysis of eosinophilic solid cystic carcinoma reveals positive staining for cytokeratin (CK)20, PAX8, vimentin, CK8/18, and AE3. However, CD117 and CK7 typically exhibit negative staining [46].

The ALK-rearranged RCC

In the WHO 2016 categorization, ALK-rearranged RCC was regarded as an emerging entity. The 2022 categorization by the World Health Organization (WHO), specifically the 5th edition, designated it as a unique category within the part about molecularly characterized RCC. Anaplastic lymphoma kinase-rearranged RCC is characterized by the occurrence of ALK gene fusion events involving various genes, such as VCL, HOOK1, EML4, CLIP1, and KIF5B, located at chromosome 2p23. These gene fusions result in the abnormal activation of ALK signaling pathways. The presence of VCL-ALK gene fusion has been observed in young individuals who have the sickle cell trait [48,49]. The diagnosis of ALK gene rearrangement can be achieved by the use of FISH or IHC techniques. The course of the disease exhibits both aggressive and patient characteristics, which can be effectively treated with targeted ALK inhibitors [50].

The ELOC-mutated RCC

This category included in the WHO 2022 classification falls within the novel category, specifically under the part of molecularly characterized renal cancer [51]. The tumour cells within this particular category have distinct, clear cell morphology, characterized by an abundance of transparent cytoplasm. Additionally, fibromuscular bands are observed at the periphery of the tumour, along with a papillary or acinar pattern. Therefore, it is typically the pathologist's role to diagnose this tumour as clear cell RCC with fibromuscular or leiomyomatous stroma. The utilization of molecular testing is necessary to identify the presence of a mutation in the ELOC gene (TCEB1) and classify the tumour as an ELOC-mutated RCC. The majority of this tumour exhibits a slow-growing nature following surgical removal, as indicated by the positive expression of CK7 in IHC [7,52].

Low-grade oncocytic tumour

The 2022 categorization by the WHO designates the low-grade oncocytic tumour as an emerging entity within the category of various oncocytic tumours of the kidney. This study highlights the morphological characteristics that lie between oncocytoma and chromophobe RCC. Typically, it manifests as a solitary, intermittent neoplasm, occasionally occurring in the advanced stages of kidney illness and tuberous sclerosis complex (TSC) [53,54].

This tumour is rather tiny in size and exhibits a mahogany brown colouration upon sectioning. Under microscopic examination, the specimen exhibits a growth pattern characterized by tubules, reticula, or trabeculae. The observed cells do not exhibit high-grade nuclear characteristics, such as increased mitosis, multinucleation, pleomorphism, and so forth. The observed characteristics include modest nuclear expansion and perinuclear clearance, as reported in previous studies [55,56]. The tumour exhibits positive antibodies for CK7 and GATA transcription factor. The stimulation of the mammalian target of rapamycin (mTOR) pathway has been observed in this particular tumour [57].

Eosinophilic vacuolated tumour

The 2022 categorization by the WHO designates the eosinophilic vacuolated tumour as an emerging entity categorized within the part of various oncocytic tumours of the kidney [58]. The tumour in question is characterized by its tiny size, solitary nature, and absence of a capsule or cystic component. Under microscopic examination, the specimen reveals a nucleus that is round to oval in shape, accompanied by cytoplasm that appears eosinophilic. Additionally, intracytoplasmic vacuoles are observed within the cytoplasm [59,60]. This neoplasm exhibits a correlation with TSC and genetic alterations in the mTOR, TSC2, and TSC1 genes. The IHC analysis reveals the presence of CD117 and cathepsin K positivity [61]. All of the key changes have been organized and shown in Table 1.

**Table 1:** Key updates in the WHO 2022 classification (5th edition) of renal tumours RCC: Renal cell carcinoma, PRCC: Papillary renal cell carcinoma, FH: Fumarate hydratase, SMARCB-1: SWI/SNF‐related matrix‐associated actin‐dependent regulator of chromatin subfamily B member 1, TFE3: Transcription factor binding to IGHM enhancer 3, TFEB: Transcription factor EB, MIT: Microphthalmia, ALK: Anaplastic lymphoma kinase, ELOC: Elongin C [11,12,20-22,27-31,34-36,46-49,51-53,58]

No.	Name of renal tumour	WHO 2016 classification	Key updates in the WHO 2022 classification
1.	PRCC	Subclassification into type 1 and type 2 recommended	Subclassification into type 1 and type 2 is NOT recommended. Morphology patterns updated, such as Warthin-like RCC and PRCC with reverse polarity.
2.	Clear cell papillary renal cell tumour	The name given to the tumour is 'clear cell papillary RCC'.	Due to its indolent behaviour and no evidence of metastasis, the name was changed from clear cell papillary RCC to clear cell papillary renal cell tumour.
3.	FH-deficient RCC	The name of the tumour in this classification was hereditary leiomyomatosis associated with RCC due to its association with uterine and cutaneous leiomyoma.	The FH protein deficiency is observed in many categories along with this one. Hence, the name was changed to FH-deficient RCC.
4.	SMARCB1-deficient renal medullary carcinoma	Due to its constant location observed at the renal medulla region, it was given the name 'renal medullary carcinoma'.	The SMARCB1 deficiency is also observed in this category. So, the name was changed to SMARCB1-deficient renal medullary carcinoma.
5.	TFE3-rearranged and TFEB-altered RCC	It was categorised together as the 'MIT family of RCC'	The MIT category no longer exists. Two separate categories were made, namely the TFE3-rearranged and TFEB-altered RCC. Due to different clinical behaviour and prognosis, TFEB-altered RCC was again categorized into TFEB-rearranged and TFEB-amplified RCC.
6.	Eosinophilic solid and cystic RCC	No such category present	It is added as a new entity under the category of 'other renal tumours'.
7.	ALK-rearranged RCC	An emerging entity in this classification.	It is added as a new entity under the category of 'molecularly defined renal carcinoma'.
8.	ELOC-mutated RCC	An emerging entity known as RCC with leiomyomatous stroma in this classification.	It is added as a new entity under the category of 'molecularly defined renal carcinoma'.
9.	Low-grade oncocytic tumours	No such category	Emerging entity
10.	Eosinophilic vacuolated tumour	No such category	Emerging entity

## Conclusions

There are several significant modifications in the WHO's 5th edition of renal tumours classification as compared to the WHO's 2016 classification. Nomenclature modifications were observed in the context of papillary and clear cell renal cancer: HLRCC nomenclature was modified to FH-deficient RCC, and renal medullary cancer has been revised to SMARCB1-deficient RCC. The MIT category no longer exists in the new classification. The TFE3-rearranged and TFEB-altered RCC are now separated. Eosinophilic solid and cystic RCC is a newly added entity under the category of ‘other renal tumours’. The ALK-rearranged RCC and ELOC-mutated RCC are classified as novel molecularly defined entities. Low-grade oncocytic tumours and eosinophilic vacuolated tumours are classified as emerging entities.
